# Temporal Changes in Jejunal and Ileal Microbiota of Broiler Chickens with Clinical Coccidiosis (*Eimeria maxima*)

**DOI:** 10.3390/ani14202976

**Published:** 2024-10-15

**Authors:** Katarzyna B. Miska, Philip M. Campos, Sara E. Cloft, Mark C. Jenkins, Monika Proszkowiec-Weglarz

**Affiliations:** 1 Animal Biosciences and Biotechnology Laboratory (ABBL), Beltsville Agricultural Research Center (BARC), United States Department of Agriculture—Agricultural Research Service (USDA—ARS), Beltsville, MD 20705, USA; philip.campos@usda.gov (P.M.C.); monika.weglarz@usda.gov (M.P.-W.); 2 Animal Sciences Department, Purdue University, West Lafayette, IN 47907, USA; scloft@purdue.edu; 3 Animal Parasitic Diseases Laboratory (ABBL), Beltsville Agricultural Research Center (BARC), United States Department of Agriculture—Agricultural Research Service (USDA—ARS), Beltsville, MD 20705, USA; mark.jenkins@usda.gov

**Keywords:** coccidiosis, *Eimeria maxima*, microbiota, broilers, 16S rRNA sequencing, gut morphology

## Abstract

Coccidiosis in broiler chickens continues to be a major disease of the gastrointestinal tract, causing economic losses to the poultry industry worldwide. The goal of this study was to generate a symptomatic *Eimeria maxima* (1000 oocysts) infection to determine its effect on the luminal and mucosal microbiota populations (L and M) in the jejunum and ileum (J and IL). Samples were taken from day 0 to 14 post-infection, and sequencing of 16S rRNA was performed using Illumina technology. Infected birds had significantly (*p* < 0.0001) lower body weight gain (BWG), higher feed conversion ratio (FCR) (*p =* 0.0015), increased crypt depth, and decreased villus height (*p* < 0.05). The significant differences in alpha and beta diversity were observed primarily at height of infection (D7). Analysis of taxonomy indicated that J-L and M were dominated by *Lactobacillus*, and in IL-M, changeover from *Candidatus Arthromitus* to *Lactobacillus* as the major taxon was observed, which occurred quicky in infected animals. LEfSe analysis found that in the J-M of infected chickens, *Lactobacillus* was significantly more abundant in infected (IF) chickens. These findings show that *E. maxima* infection affects the microbiota of the small intestine in a time-dependent manner, with different effects on the luminal and mucosal populations.

## Simple Summary

Coccidiosis affects broiler chickens, causing economic losses since infected chickens grow less efficiently than healthy birds. Coccidiosis is caused by a parasite *Eimeria* that infects the gastrointestinal tract. In the current study, changes in the bacteria of the jejunum and ileum following infection with *Eimeria maxima* were determined. Samples were taken between 0 and 14 days post-infection. The infection resulted in decreased body weight gain, increased feed conversion ratio, compromised gut morphology, and decreased plasma carotenoid levels. Microbiota composition was determined by sequencing a portion of the 16s rDNA gene. The results indicate that infection affected the diversity within and between bacterial communities, particularly at the height of infection. In samples from infected birds, species of bacteria that can be opportunistic pathogens were more abundant than in healthy birds. In uninfected birds, bacteria that produce short-chain fatty acids and are associated with improved growth were more abundant. *Eimeria maxima* affects the ecology of the small intestine by disrupting its integrity and changes the types of bacteria present to those that could be opportunistic pathogens. Coccidiosis is a complex gastrointestinal disease, and understanding its effects on the host will help find effective control methods in the future.

## 1. Introduction

Consumption of broilers has been increasing steadily since the modern chicken industry began selecting birds for rapid weight gain in the 1960s. In 2024, it is estimated that the per capita consumption of meat derived from broilers will reach 100 pounds in the USA (Per Capita Consumption of Poultry and Livestock, 1960 to Forecast 2024, in Pounds—National Chicken Council). Chicken meat is a good source of protein, and its price is lower compared to other options such as pork and beef (Wholesale and Retail USDA Prices for Chicken, Beef, and Pork—National Chicken Council), making it an affordable alternative. Though broiler chicken meat is in demand and its growth is expanding, there are challenges to maintaining high production while maintaining affordable cost. Modern broilers are predominantly grown in intensive farming operations where they are exposed to many types of pathogens. One of the most prevalent diseases is coccidiosis, which is caused by protozoan parasites belonging to genus *Eimeria* (Mesa-Pineda et al., 2021) [1]. There are at least seven described species; however, other cryptic species have been recently described (Blake et al., 2021) [2]. Coccidiosis in broiler chickens results in reduction of growth parameters since *Eimeria* infects the intestinal tract and causes diarrhea, inappetence, lethargy, and in some cases mortality (Mesa-Pineda et al., 2021) [1]. Currently coccidiosis is controlled largely by vaccines and chemotherapeutic agents, but new chemotherapeutic agents have not been developed recently (Ahmad et al., 2024) [3].

*Eimeria maxima* is one of the established species that causes coccidiosis in chickens. It infects the small intestine (jejunum, ileum, and can appear in the duodenum) (Noack et al., 2019) [4]. The *Eimeria* life cycle is initiated when a chicken ingests sporulated oocysts, which are excysted releasing sporozoites into the intestine. The excysted sporozoites infect epithelial cells (other cell types can be infected as well) and undergo several stages of asexual reproduction until they develop sexual stages (micro- and macrogametocytes) that undergo fertilization followed by meiosis to produce oocysts that are excreted in the feces. To become infectious, the oocysts must mature by sporulating in the litter. The entire life cycle is completed in 6–7 days (depending on the species), with clinical symptoms appearing as early as four days post-infection (PI) (Martins et al., 20222) [5]. Much of the research concerning *Eimeria* has been focused on gathering data at the height of infection; however, changes in the intestine occur prior, beginning with the invasion of sporozoites, and persist past the average of seven days needed to complete the life cycle (Cloft et al., 2023; Elsasser et al., 2018; Rothwell et al., 1995) [6,7,8], but it is unclear when during the infection the microbiota becomes altered. It has previously been shown that changes to the microbiota can take place in the lumen and mucosa of the small and large intestine at the height of infection and remain up to 14 days PI (Campos et al., 2023, 2024) [9,10]. The aim of the current study was to establish and measure a clinical *E. maxima* infection and investigate the changes in bird growth parameters, gut morphology, and microbiota through 16S rRNA sequencing in the small intestine. Because the primary target of *E. maxima* infection is the small intestine, changes in microbiota were measured that are most likely caused directly by the pathogen. Many studies measuring microbiota populations sample intestinal contents, even though it has been observed that the populations of bacteria that reside in the lumen of the gut are distinct from those that adhere to the gut epithelium (mucosal population) (Borda-Molina et al. 2018; Campos et al., 2022; Proszkowiec-Weglarz, 2022) [11,12,13]. It was hypothesized that *E. maxima* infection would disrupt the microbiota most severely during the height of infection, and that the changes of the luminal and mucosal microbiota would differ.

## 2. Materials and Methods

### 2.1. Animal Husbandry and Tissue Sampling

All animal care procedures were approved by the Institutional Animal Care and Use Committee (IACUC, protocol #18-025) of the Beltsville Agricultural Research Center (BARC). Two hundred and eighty-eight Ross 708 male broiler hatchlings were obtained from Longnecker’s Hatchery (Elizabethtown, PA, USA) and placed into 1.00 m^2^ open-top wire brooder pens (approximately 25 chicks per pen). At 19 days of age, all birds were moved to 72 cages (Alternative Designs, Siloam Springs, AR, USA) with 4 birds per pen. A corn–soybean-based diet (approximately 24% crude protein, crumble) and water were provided to chicks ad libitum for the duration of the study. One-half of the birds (144 birds) were infected (IF) with 1 × 10^3^ *E. maxima* oocysts (USDA APU1 isolate) in a volume of 1.0 mL per bird by oral gavage at 21 days of age. The remaining 144 birds were sham-infected with water (control [C]). A total of 36 pens of C birds and 36 pens of IF birds were housed in a single room, on opposite sides to prevent cross-transmission.

At six time points (0, 3, 5, 7, 10, and 14 days PI), birds and feed were weighed to calculate body weight (BW), body weight gain (BWG), feed intake (FI), and feed conversion ratio (FCR). For this study, the pen was treated as an experimental unit, with 6 replicates per time point. For the BW, BWG, FI, and FCR, the average value per cage was determined from all 4 birds/cage. Blood samples were collected via cardiac puncture from one bird per cage following cervical dislocation into an EDTA tube and centrifuged at 2000× *g* at 4 °C to collect plasma for carotenoid measurements using previously described methods (Allen, 1987) [14]. The concentration of plasma carotenoids was used to evaluate the level of *E. maxima* infection. For tissue morphology, a 2–3 cm segment of the jejunum was dissected, rinsed with sterile PBS, and placed into neutral buffered formalin for later histological analysis.

For luminal contents and mucosal microbiota sampling, the jejunum and ileum of one bird per cage (6 replicates per treatment) were dissected, and the jejunal and ileal contents (J-C and IL-C) and scrapings (J-M and IL-M) were collected. The contents of the jejunum and ileum were collected by squeezing the digesta by hand into cryovials. The intestine was then placed in sterile ice cold 1× PBS, opened with scissors longitudinally and rinsed to remove any contents still adhering to the mucosa. The mucosa was then scraped using a microscope slide and the tissues were also placed into cryovials. Isolated specimens were snap frozen in liquid nitrogen and stored at −80 °C until bacterial DNA isolation.

### 2.2. Identification of Stem Cells and Morphological Measurements

Jejunum segments collected for histological analysis were transferred to 70% ethanol and kept at 4 °C until they were embedded in paraffin (StageBio, Mount Jackson, VA, USA). Formalin-fixed, paraffin-embedded jejunum tissues were utilized for in situ hybridization of olfactomedin 4 (Olfm4; NM_001040463.1) as described in Cloft et al. (2023) [6]. Olfm4 is an intestinal stem cell marker that defines the functional crypts as opposed to the visible crypt, providing a more objective measure (Cloft et al., 2023) [6]. Images were captured with a Nikon Eclipse 80i microscope with a DS-Ri1 digital camera (Nikon Instruments, Inc., Melville, NY, USA) at 40× magnification. Morphological measurements of crypt depth (CD) and villus height (VH) were taken with Image J software (version 15.0) from the National Institutes of Health (Bethesda, MD, USA). Approximately 75 crypts and 30 villi were measured on 4 birds per treatment timepoint.

### 2.3. DNA Isolation and 16s rRNA Library Preparation

DNA was extracted from J-C, J-M, IL-C, and IL-M and was evaluated as described previously (Campos et al., 2022) [12]. The 16S rRNA gene amplicon libraries were generated using the workflow and chemistry supplied by Illumina (Illumina, Inc., San Diego, CA, USA), and PCR primers (Forward: 5′-TCGTCGGCAGCGTCAGATGTGTATAAGAGACAGCCTACGGGNGGCWGCAG-3′ and Reverse: 5′-GTCTCGTGGGCTCGGAGATGTGTATAAGAGACAGGACTACHV GGGTATCTAATCC-3′) targeted the V3-V4 variable region of the 16S gene (Darwish et al., 2021) [15]. Amplicon PCR was followed by index PCR followed by amplicon cleaning, as described previously (Campos et al., 2022) [12]. The concentration and quality of the amplicons were determined using QIAxcel DNA Hi-Resolution cartridge, proprietary QIAxcel ScreenGel software (version 1.6.0, and QIAxcel Advanced System (Qiagen, Germantown, MD, USA) per manufacturing instructions. The pooled (96 barcoded amplicons) DNA library (4 nM) and PhiX control v3 (Illumina, Inc., San Diego, CA, USA) (4 nM) were denatured with 0.2 N NaOH (Sigma-Aldrich, Corp., St. Louis, MO, USA) and diluted to a final concentration of 4 pM. The library was mixed with PhiX control (20% *v*/*v*), and pair-end 2 × 300 bp sequencing was performed using the Illumina MiSeq platform and a MiSeq Reagent Kit v3 (Illumina, Inc.).

### 2.4. Bioinformatics and Data Processing

Quantitative Insight Into Microbial Ecology (QIIME) software package 2 (version 2023.2, http://qiime2.org accessed on 3 October 2024) (Bolyen et al., 2019) [11] was used to perform quality control and analysis of the sequence reads as described before (Campos et al., 2022) [12]. Raw fastq files were demultiplexed using q2-demux and quality filtered and denoised with DADA2 via q2-dada2 (Callahan et al., 2016) [16]. Sequences were trimmed with DADA2 where remaining base positions had a median Phred quality score of 30 or above (see Table 1 for truncation parameters). Representative sequence sets containing DADA2 amplicon sequence variants (ASVs) were used for taxonomy classification. MAFFT (Katoh et al., 2002) [17] was used for multiple sequence alignment, and FastTree2 (Price et al., 2010) [18] was used to generate phylogenetic trees. Naïve Bayesian classifier was used for taxonomic classification against the SILVA version 138 database (Quast et al., 2012) [19], which was chosen over the Greengenes 2013 database for its larger size and more up-to-date taxonomy (Balvočiūtė et al., 2017; Campos et al., 2022) [12,20]. For this step, RESCRIPt pre-formatted SILVA 99% reference sequences, and taxonomy files were obtained from the QIIME 2 Data Resources page (https://docs.qiime2.org/2023.2/data-resources/ accessed on 3 October 2024), as RESCRIPt reduces inconsistencies by removing duplicate sequences that are assigned different taxonomies (Robeson et al., 2021) [21]. Data were rarefied to sequencing depths (Table 1) using alpha rarefaction plots to consider where bacterial diversity would be maintained for the calculation of alpha and beta diversities. Alpha diversity indices (observed features (ASVs), Shannon’s diversity index, Pielou’s Evenness (evenness), and Faith’s Phylogenetic Diversity (richness)) were obtained through QIIME 2 package. Analysis of beta diversity was performed by QIIME2 employing unweighted (Lozupone et al., 2005) [22] and weighted UniFrac (Lozupone et al., 2007) [23] distance metrics. The linear discriminant analysis (LDA) effect size (LEfSe) algorithm was used to identify taxa with significant differential abundance between C and IF birds (Segata et al., 2011) [24]. Genus-level feature tables were converted to relative abundance and analyzed at the Huttenhower Lab Galaxy web server (http://huttenhower.sph.harvard.edu/galaxy accessed on 3 October 2024) using the default parameters (LDA threshold of 2.0). Phylogenetic Investigation of Communities by Reconstruction of Unobserved States 2 (PICRUSt2 version 2.4.2) software was used to predict functional abundances based on marker gene sequences (Douglas et al., 2020) [25]. The MetaCyc Metabolic Pathways Database (Caspi et al., 2020) [26] was used to produce functional abundance data, and the data were analyzed and visualized using STAMP 2.1.3 (Parks et al., 2014) [27] to determine the biological relevance of features.

Visualizations for alpha diversity comparisons and beta diversity PCoA were produced in R 4.3.2 (R Core Team, 2023) [28] using the packages QIIME2R 0.99.35 (Bisanz et al., 2020) [29] to import QIIME 2 PCoA results and tidyverse 2.0.0 (Wickham et al., 2020) [30] for data wrangling with dplyr (Wickham et al., 2023) [31] and figure production with ggplot2 (Wickham 2016) [32]. To produce taxonomic bar plot visualizations, samples were merged into groups (infection status × time point) using the R package phyloseq version 1.48.0 (McMurdie and Holmes, 2013) [33], and taxonomic data were processed at the genus level using microViz version 0.12.5 (Barnett et al., 2021) [34].

### 2.5. Statistical Analysis

Performance data (BW, BWG, FI, FCR) and plasma carotenoid concentration were analyzed by 2-factor ANOVA considering time post-infection and infection status as main factors via JMP v15.0 (SAS Institute Cary, NC, USA). When significant ANOVA results were observed, Tukey’s honestly significant difference test was conducted for mean separation.

Morphological data were unable to be transformed to meet conditions of normal distribution due to unequal variances; therefore, data were analyzed using the nonparametric Welch’s one-way test for the effect of infection status at each timepoint.

Differences between alpha diversity indices were tested using the Kruskal–Wallis test (QIIME2). The difference in community structure based on groups (infection status × time point) were statistically tested by non-parametric multivariate ANOVA (PERMANOVA) with 999 permutations using QIIME2, and distances between samples were visualized with PCoA. Statistical significance was established at *p* ≤ 0.05 for all analyses.

## 3. Results

### 3.1. Parameters Associated with Infection and Growth

The performance parameters of birds included in this study are shown in Table 2. These include body weight (BW), body weight gain (BWG), feed intake (FI), and feed conversion ratio (FCR). Statistical analysis of significant differences within, as well as between, main effects (infection status and age) were determined by analysis of variance. The BW of birds increased through time (*p <* 0.0001), and IF birds weighed less than C birds (*p <* 0.0246). The BWG of birds increased through time (*p <* 0.0001), and IF birds gained less weight than C birds (*p <* 0.0001). The FI did not differ significantly between C and IF birds (*p =* 0.33) but increased significantly through time (*p <* 0.0001). There was a significant interaction between the main effects (*p =* 0.0487) in FCR.

**Figure 1:**
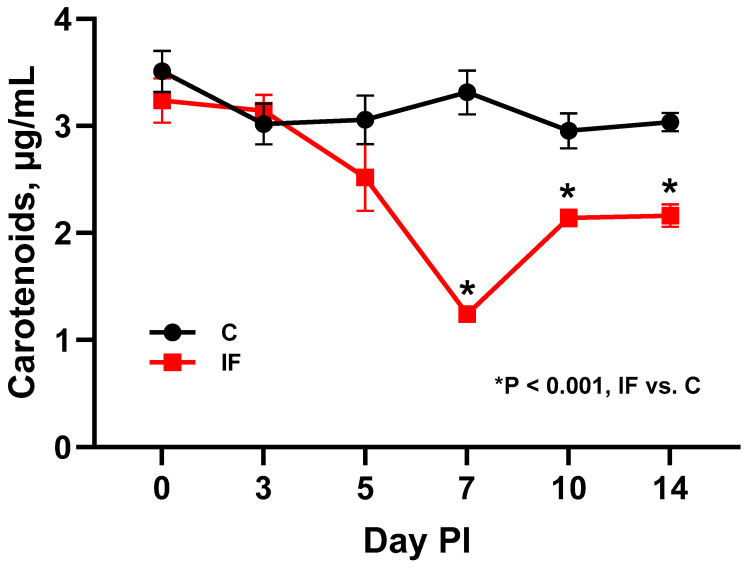
Concentration of plasma carotenoids in control (C) and *Eimeria maxima* (IF)-infected chickens (day PI, day post-infection; mean ± SE).

To further ensure that an *E. maxima* infection was present, the level of plasma carotenoids (Allen, 1987) [14] was measured at each of the six time-points and is shown in Figure 1. There was a significant decrease in plasma carotenoids in IF birds (*p* < 0.001) beginning at 7 days PI. The carotenoids increased at days 10 and 14 PI but were still significantly lower than those in C birds.

### 3.2. Gut Morphology

**Figure 2:**
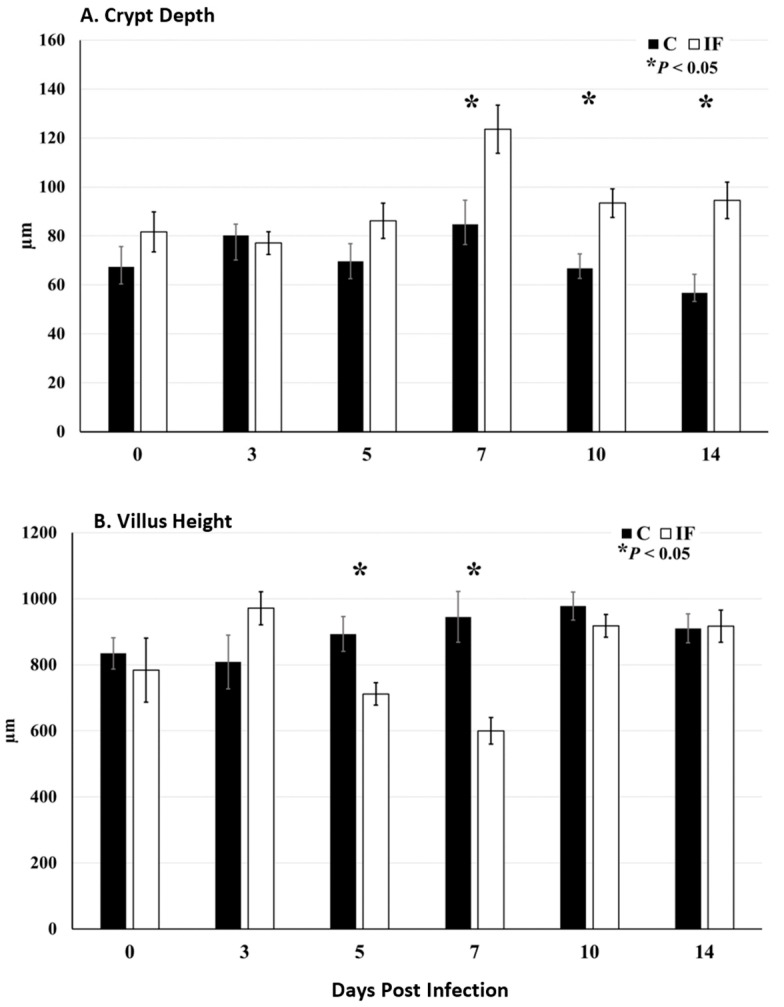
Crypt elongation following infection with *Eimeria maxima*. Expression of *Olfm4* mRNA by in situ hybridization in the jejunum of broiler chickens that were infected at 21 d of age with either 1000 *E. maxima* oocysts (IF) or sham infected with sterile water (C) and sampled at 0, 3, 5, 7, 10, and 14 d post-infection (PI). All tissues were counterstained with 50% hematoxylin. Images were captured at 40× magnification (n = 4). (**A**) Crypt depth and (**B**) villus height was measured on jejunal sections stained for *Olfm4* by in situ hybridization. Measures were analyzed by infection status using the nonparametric Welch’s one-way test. Significances (*p* < 0.05) are indicated by asterisks (*).

The crypt depth of the jejunum of IF birds increased significantly beginning at 7 days PI and remained significantly elevated through the remainder of the study (*p* ≤ 0.0051) compared to C birds (Figure 2A). At days 5 and 7 PI, the villus height of IF birds was significantly decreased (*p* = 0.0074) compared to C birds (Figure 2B).

### 3.3. Sequencing Summary

The summary of the sequences produced during this study is shown in Table 1. The sequence depth for rarefaction was lower in the mucosa samples (5658 J-M and 13,067 IL-M) compared to the luminal contents (J-C 27,462 and IL-C 49,158).

### 3.4. Alpha and Beta Diversity of the Microbiota of the Small Intestine

**Figure 3:**
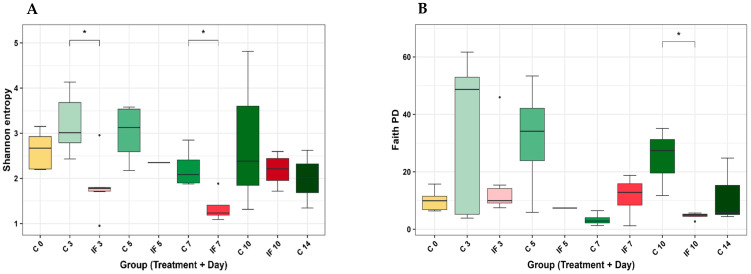
Effect of *Eimeria maxima* infection at days 0, 3, 5, 7, 10, and 14 post-infection (PI) on the alpha diversity indices (**A**) Shannon’s entropy and (**B**) Faith’s PD of the mucosal bacterial populations of the jejunum (J-M). Non-infected birds = C, infected birds = IF. Significant (*p* < 0.05) differences are indicated by asterisks (*).

**Figure 4:**
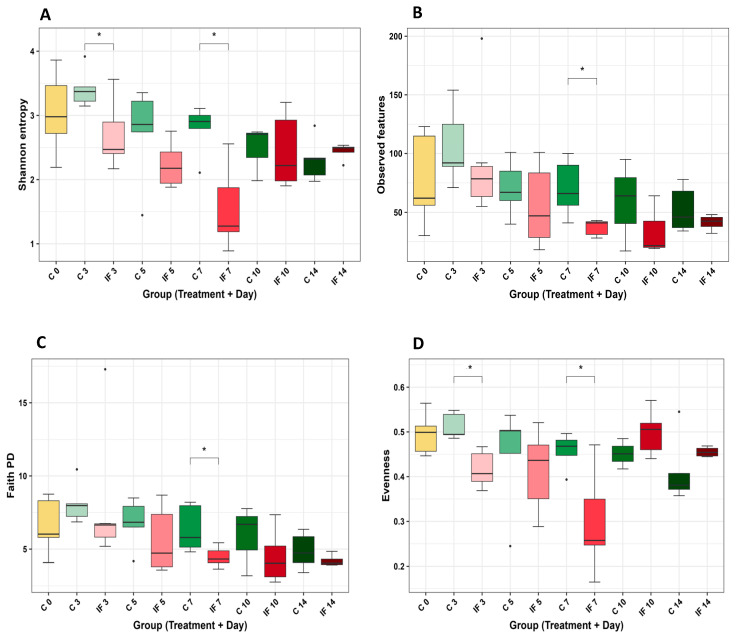
Effect of *Eimeria maxima* infection at days 0, 3, 5, 7, 10, and 14 post-infection (PI) on the alpha diversity indices (**A**) Shannon entropy, (**B**) observed features, Faith’s PD (**C**), and Evenness (**D**) of the luminal bacterial populations of the ileum (IL-C). Non-infected birds = C, infected birds = IF. Significant (*p* < 0.05) differences are indicated by asterisks (*).

There were no significant differences in alpha diversity in either the J-C or IL-M samples, and the significant differences in J-M are shown in Figure 3. Shannon’s entropy was significantly lower in the IF samples at days 3 (*p* = 0.02) and 7 PI (*p* = 0.04) (Figure 3A), and Faith’s PD was significantly lower in the IF samples at day 10 PI (*p* = 0.03). The significant differences in alpha diversity of IL-C are shown in Figure 4. Shannon’s entropy and Evenness were significantly lower between C and IF samples and at days 3 (*p* = 0.04) and 7 (*p* = 0.02) (Figure 4A and Figure 4D, respectively). Observed features (*p* = 0.04) and Faith’s PD (*p* = 0.047) were significantly lower between C and IF samples on day 7 PI (Figure 4B and Figure 4C, respectively).

**Figure 5:**
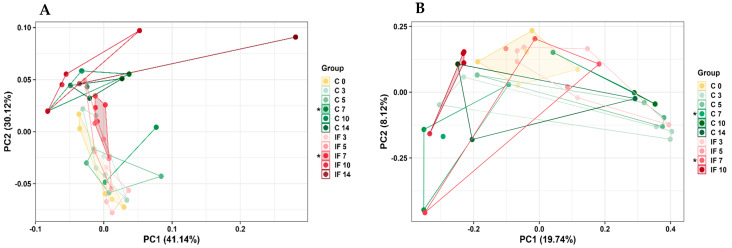
Effect of *Eimeria maxima* at days 0, 3, 5, 7, 10, and 14 post-infection (PI) on the beta diversity of jejunal luminal (**A**) (J-C) and jejunal mucosa (**B**) (J-M) bacterial populations using the principal coordinate analysis (PcoA) based on the weighted (**A**) and unweighted UniFrac (**B**) distances between groups. Non-infected birds = C, infected birds = IF. Significant (*p*  <  0.05) differences are indicated by asterisks (*).

**Figure 6:**
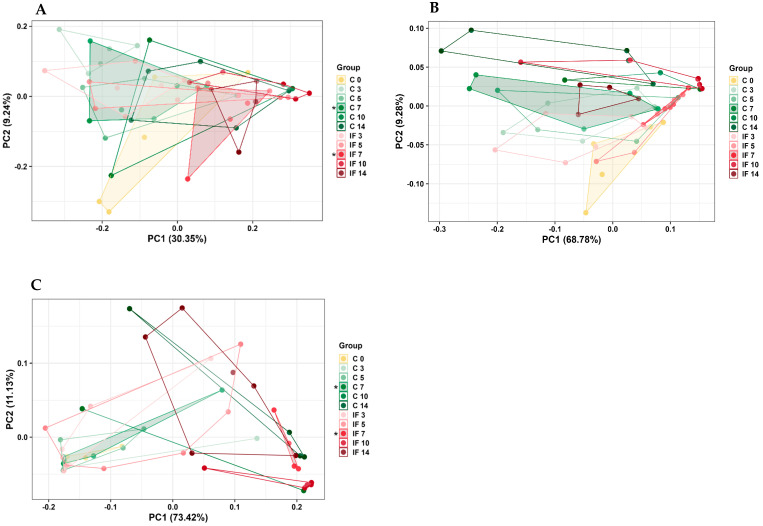
Effect of *Eimeria maxima* at days 0, 3, 5, 7, 10, and 14 post-infection (PI) on the beta diversity of ileal luminal (**A**,**B**) (IL-C) and ileal mucosa (**C**) (IL-M) bacterial populations using the principal coordinate analysis (PcoA) based on the weighted (**A**,**C**) and unweighted UniFrac (**B**) distances between groups. Non-infected birds = C, infected birds = IF. Significant (*p* < 0.05) differences are indicated by asterisks (*).

Beta diversity measures the amount of variability between bacterial communities (Figure 5 and Figure 6) and was measured using phylogenetic-based unweighted (UWT) or weighted Uni-Frac (WT) methods. The UWT Uni-Frac analysis of J-C was not significant, but WT Uni-Frac of J-C was significantly different (*p* = 0.04) on day 7 PI between C and IF (Figure 5A). Conversely, the WT UniFrac analysis of J-M was not significant, but UWT UniFrac of J-M was significantly different (*p* = 0.03) on day 10 PI between C and IF (Figure 5B).

The beta diversity in both IL-C and IL-M samples was significantly different on day 7 PI (*p* = 0.02 and 0.01, respectively) (Figure 6A,B). For IL-M, the UWT Uni-Frac was not significant, but significant differences were measured using WT Uni-Frac (*p* = 0.02) between C and IF at day 7 PI (Figure 6C). In general, the results of beta diversity analysis showed significant differences between bacterial communities at day 7 PI that corresponded to height of *E. maxima* infection.

### 3.5. Taxonomy

**Figure 7:**
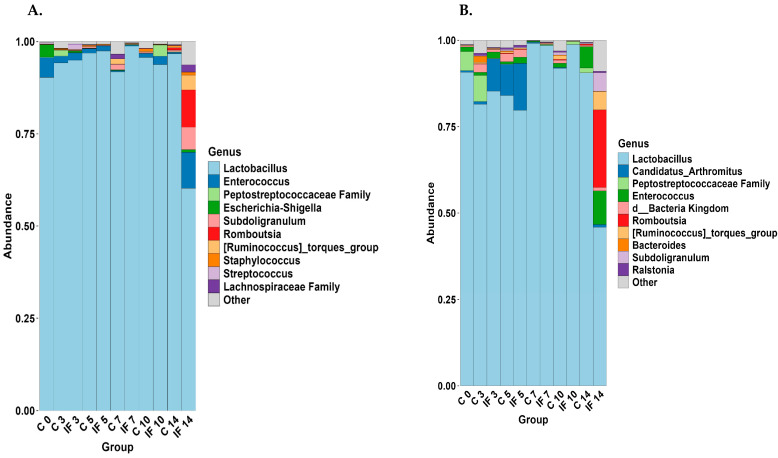
Effect of *Eimeria maxima* at days 0, 3, 5, 7, 10, and 14 post-infection (PI) on relative bacterial abundance (%) at the genus level in the (**A**) jejunal lumen (J-C) and (**B**) jejunal mucosa (J-M). Non-infected birds = C, infected birds = IF.

**Figure 8:**
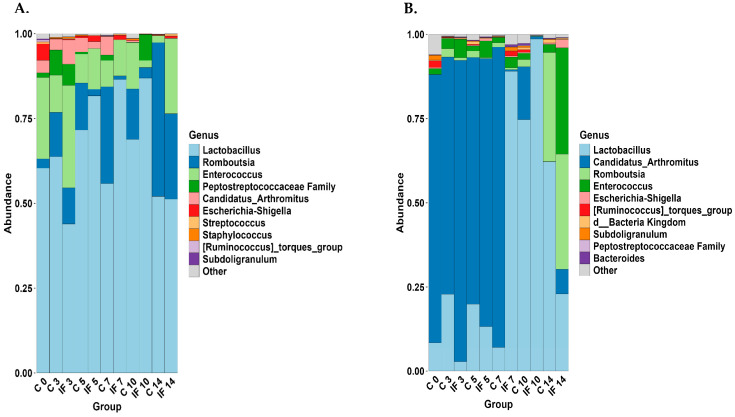
Effect of *Eimeria maxima* at days 0, 3, 5, 7, 10, and 14 post-infection (PI) on relative bacterial abundance (%) at the genus level in the (**A**) ileal lumen (IL-C) and (**B**) ileal mucosa (IL-M). Non-infected birds = C, infected birds = IF.

The 11 most abundant taxa (% of the genera present) are shown for J-C and J-M (Figure 7) and IL-C and IL-M (Figure 8). The taxa present between C and IF in the J (both C and M) (Figure 7A,B) at corresponding days were very similar, except for IF at D14, where the abundance of *Lactobacillus* was lower. In the jejunum (both C and M), the most abundant genus was *Lactobacillus*. In all samples with the exception of those from IF birds at day 14, it made up more than 75% of the genera present. The taxa that were present in J-M but not J-C were *Escherichia–Shigella*, *Staphylococcus*, *Streptococcus*, and the *Lachnospiraceae*. On the other hand, *Candidatus Arthromitus*, *Enterococcus*, Bacteria kingdom (which includes unclassified sequences that could not be classified beyond the kingdom), and *Ralstonia* were present in J-M but not J-C. Of the taxa present in IL-C (Figure 8A), *Lactobacillus* was more abundant in IF samples at 5, 7, and 10 days PI. *Streptococcus* and *Staphyloccocus* were present in IL-C but not IL-M. On the other hand, unclassified bacteria and *Subdoligranulum* were found in IL-M but not IL-C. In the IL-M, the level of *Lactobacillus* was higher in samples from IF7, C10, IF10, and C14.

### 3.6. Differentially Abundant Taxa

**Figure 9:**
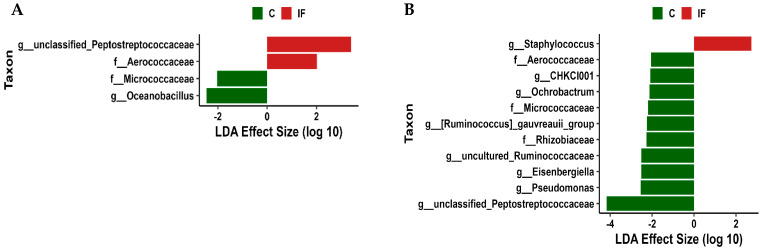
Effect of *Eimeria maxima* on differentially abundant bacterial genera as determined by linear discriminant analysis (LDA) effect size (LEfSe) analysis in jejunal lumen (J-C, (**A**)) and mucosal (J-M, (**B**)) bacterial populations. C—uninfected chickens, IF—infected chickens.

**Figure 10:**
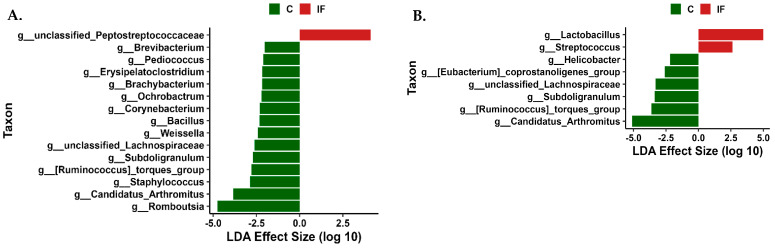
Effect of *Eimeria maxima* on differentially abundant bacterial genera as determined by linear discriminant analysis (LDA) effect size (LEfSe) analysis in ileal lumen (IL-C, (**A**)) and mucosal (IL-M, (**B**)) bacterial populations. C—uninfected chickens, IF—infected chickens.

LEfSe analysis was performed to determine differentially abundant taxa in the J-C and J-M (Figure 9) and in the IL-C and IL-M (Figure 10). In the J-C, bacteria belonging to the genus unclassified Peptostreptococcaceae and family Aerococcaceae were more abundant in samples from IF animals, while family Micrococcaceae and genus *Oceanobacillus* were more abundant in C samples (Figure 9A). In the J-M, members of the unclassified genus *Staphyloccocus* were more abundant in IF samples, and eight genera (CHKCI001, *Ochrobactrum*, [*Ruminococcus*] *gauvreauii* group, uncultured Ruminococcaceae, *Eisenbergiella*, *Pseudomonas*, unclassified Peptostreptococcaceae) and three families (Aerococcaceae, Rhizobiaceae, and Micrococcaceae) were more abundant in C samples (Figure 9B).

In the IL-C, members of the unclassified genus *Staphyloccocus* were more abundant in IF samples, and 12 different genera (shown in Figure 10A) were more abundant in C samples. In the IL-M, the genus *Lactobacillus* and *Streptococcus* were more abundant in IF samples, and the genera *Helicobacter*, [*Eubacterium*] *coprostanoligenes* group, unclassified *Lachnospiraceae*, *Subdilogranulum*, [*Ruminoccocus*] *torques* group, and *Candidatus Arthromitus* were more abundant in C samples (Figure 10B).

### 3.7. Analysis of Predicted Functional Processes

**Figure 11:**
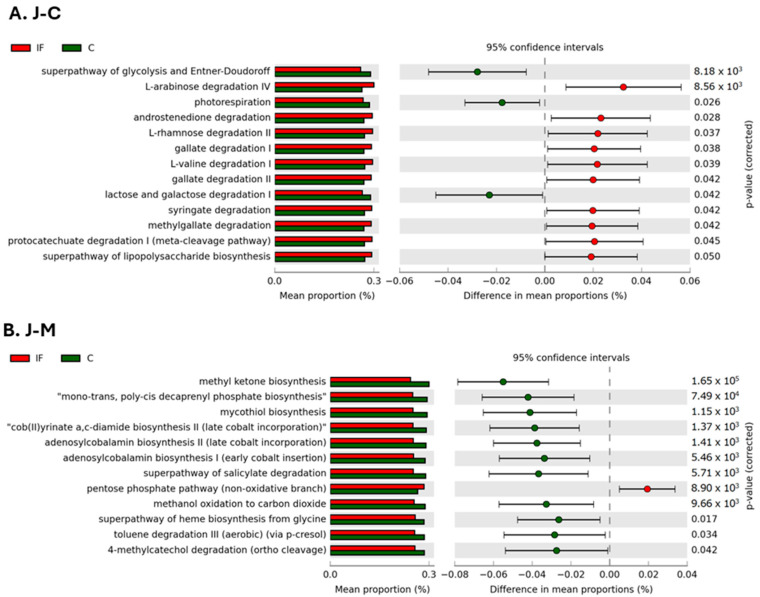
Effect of *Eimeria maxima* infection on mean proportion (%) of predicted MetaCyc pathways (up to top 21 pathways shown) in the jejunal luminal (J-C) (**A**) and jejunal mucosal (J-M) (**B**) populations.

The metabolic pathways that were predicted to be significantly different between IF and C samples collected from J-C and J-M are shown in Figure 11A and Figure 11B, respectively. In C samples from J-C, the glycolysis and Entner–Doudoroff superpathway, photorespiration, and lactose and galactose degradation were predicted to be in significantly greater abundance (Figure 11A). In IF samples from J-M, the L-arabinose, androtestosterone, L-rhamnose II, gallate I and III, L-valine I, syringate, methylgallate, and protocatechuate I degradation pathways, as well as the superpathway of lipopolysaccharide biosynthesis, were in significantly greater abundance compared to C samples (Figure 11A).

In C samples from J-M, the methyl ketone, mono-trans, poly-cis decaprenyl phosphate, mycothiol, cob(II)yrinate a,c-diamide II, adenosylcabalamin I and II, and superpathway of heme from glycine biosynthesis, as well as the superpathway of salicylate degradation, toluene degradation III, 4-methylcatechol degradation, and methanol oxidation to carbon dioxide, were in greater abundance compared to IF samples (Figure 11B). Only the pentose phosphate pathway was predicted to be in significantly greater abundance in IF samples (Figure 11B).

**Figure 12:**
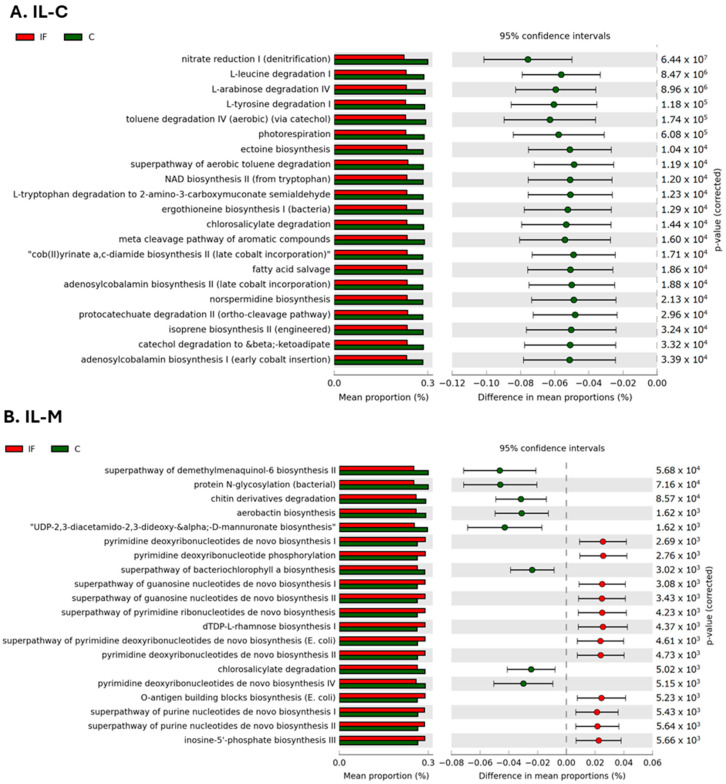
Effect of *Eimeria maxima* infection on the mean proportion (%) of predicted MetaCyc pathways (up to top 21 pathways shown) in the ileal luminal (IL-C) (**A**) and ileal mucosal (IL-M) (**B**) populations.

The metabolic pathways that were predicted to be significantly different between IF and C samples collected from IL-C and IL-M are shown in Figure 12A and Figure 12B, respectively. In the IL-C, 21 pathways were predicted to be in greater abundance in C samples, including nitrate reduction, L-leucine I, L-arabinose IV, L-tyrosine I, toluene IV, superpathway of toluene degradation, chlorosalicylate, protocatechuate II, L-tryptophan, and catechol degradation, as well as photorespiration, ectoine, NAD II, ergothioneine, cob(II)yrinate a,c-diamide II, adenosylcobalamin I and II, isoprene II and norspermidine biosynthesis, fatty acid salvage, and the meta cleavage pathway of aromatic compounds, compared to IF samples (Figure 12A). No metabolic pathways were predicted to be in greater abundance in IF samples.

In the IL-M, eight metabolic pathways were predicated to be in greater abundance in C samples, namely, chitin derivatives and chlorosalicylate degradation, protein N-glycosylation, aerobactin, D-mannuronate biosynthesis, superpathway of bacteriochlorophyll a biosynthesis, superpathway of demethylmenaquinol-6 biosynthesis II, and pyrimidine deoxyribonucleotides de novo biosynthesis IV (Figure 12B). In the IL-M, 12 metabolic pathways were predicted to be in greater abundance in IF samples in comparison to C samples: pyrimidine deoxyribonucleotide phosphorylation, pyrimidine deoxyribonucleotides de novo biosynthesis I and II, superpathway of guanosine nucleotides de novo biosynthesis I, superpathways of guanosine nucleotides de novo synthesis I and II, superpathway of pyrimidine deoxyribonucleotides de novo synthesis, superpathways of purine nucleotides de novo synthesis I and II, O-antigen building blocks biosynthesis, dTDP-L-rhamnose biosynthesis I, and inosine-5′-phosphate biosynthesis III (Figure 12B).

## 4. Discussion

In the current study, a clinical *E. maxima* infection was produced that affected the production parameters (BWG and FCR), plasma carotenoid levels, and gut morphology of the jejunum. The course of the infection was tracked, beginning on the day of infection and continuing to 14 days PI. The biggest effects on the performance parameters were observed on day 7 PI, which is standard with the previously obtained results of this *E. maxima* strain (Hansen et al., 2021; Jenkins et al., 2017) [35,36]. Measuring the levels of carotenoids in the plasma has been a useful tool in determining the level of *Eimeria* infection in the species that invade the small intestine. The levels of carotenoids in the plasma decrease following infection presumably due to decreased gut integrity where these compounds “leak” from the plasma via the compromised gut (Conway et al., 1993; Fetterer et al., 2015; Sakkas et al., 2018) [37,38,39]. Measuring the villus height and crypt depth is another way of determining presence of infection, since *Eimeria* causes a decrease in the height of the villi and an increase in the depth of the crypts (Fernando and McCraw, 1973) [40]. This was also observed at the height and recovery phase (10–14 days PI) of this experimental infection.

The major goal of this study was to determine the effects of a clinical *E. maxima* infection on the luminal and mucosal populations of the microbiota in the small intestine (the target infection site of *E. maxima*) over the two-week period. Most studies concentrate on measuring infection effects at the height of infection around days 5–7 PI (depending on species) and predominantly measure these populations in the gut contents or the excreta (Choi and Kim, 2022; Zhou et al., 2020) [41,42,43]. The small intestine is the site of nutrient absorption, and it also houses a robust population of microbiota that can be affected by *Eimeria* infection. Sufficient sequencing depth was obtained for the analysis; however, the mucosal scrapings generated fewer sequences compared to the luminal contents. This is not surprising since much less material is obtained during scraping of the mucosa, and this can also reflect that less bacteria occupy the mucosa since these are the populations that adhere to the gut epithelium (Borda-Molina et al., 2018) [13].

Significant differences in alpha diversity (within a population) were detected in the population of the jejunum scrapings and ileum contents. These differences were detected as early as day 3 PI and as late as day 10 PI. In samples from infected birds, the alpha diversity was lower compared to controls, and by day 14 PI, the populations were once again similar. By day 14 PI, the birds are in the recovery phase from coccidiosis, even though oocysts can still be shed in the feces by day 11 PI (Cha et al., 2018) [44]. It has previously been reported that coccidiosis can decrease different metrics of alpha diversity and results in a decrease in low-abundance species in the ceca (Stanley et al., 2014; Wu et al., 2014) [45,46]. However, in a study where low doses of mixed *Eimeria* species were used for infection, no significant changes in alpha diversity were observed (Leung et al., 2019) [47]. In a previous study, investigating the supplementation of feed with short-chain fatty acid, we used the same strain of *E. maxima* (APU1) and dose (1 × 10^3^ oocysts) and found that the alpha diversity of the ileum contents was the most affected (Proszkowiec-Weglarz et al., 2020) [48], which was corroborated in the current study.

Beta diversity measures the differences between microbial communities, and in the current study differences were observed in both the ileum and jejunum, primarily at the height of infection, when the effect of parasite invasion and development/replication is the highest. The effects of *E. maxima* APU1 strain infection on beta diversity in the ileum and ceca (luminal and mucosal populations) was previously reported at days 7 and 10 PI, and it was found that interactions among the main effects were observed (age, infection, tributyrin supplementation); however, in the current study, the effect of infection was the most prominent. Even though the same strain of *E. maxima* was used in Ross 308 chickens using the same poultry facilities, differences were present. This highlights the concept that bacterial populations of the gut are highly dynamic and affected by many factors, underlining the necessity for independent reproduction of experiments to truly define these populations.

The taxonomic analysis showed that most of the taxa present in the jejunum (both L and M) are made up of *Lactobacillus*. Previously, this was also observed in birds of the same age and breed but infected with *E. acervulina* (Campos et al., 2023) [9] and that *Lactobacillus* dominated both the lumen of the jejunum as well as the duodenum; however, in the mucosa, *Lactobacillus* was not the major component. It is possible that sampling methodology affected the current results; however, further investigation of the make-up of the jejunal microbiota is warranted. While the taxa of the IL-L were dominated by *Lactobacillus*, in the IL-M, a changeover from majority *Candidatus Arthromitus* to *Lactobacillus* was observed over the course of the study. This changeover was more rapid in IF animals, and the presence of both these taxa have been associated with the growth performance of chickens (Markova et al., 2024) [49].

LEfSe analysis was conducted to determine the differential abundance of bacteria between C and IF samples. Surprisingly, only a total of members of six genera and families were differentially abundant in IF samples. *Lactobacillus,* which is a beneficial probiotic genus, was more differentially abundant in the mucosal population of the IL. We have previously found that *Lactobacillus* was more differentially abundant in the ceca (both mucosal and luminal populations) and ileum (mucosa) of birds infected with *E. acervulina*, *E. tenella*, and necrotic enteritis (infected with *E. maxima* and *Clostridium perfringens*) (Campos et al., 2022; Campos et al., 2024; Latorre et al., 2018; Miska et al., 2023) [10,12,50,51]. An in vitro infection model (Tierney et al., 2004) [52] showed that indigenous *Lactobacillus* species inhibited the invasion of *E. tenella* sporozoites, and therefore it is perplexing that species that may be inhibitory to *Eimeria* invasion are more abundant in infected birds. Markova et al. (2024) [49] recently noted that the presence of many genera of lactic-acid-producing bacteria are often contradictory as to expectation, and in many cases, *Lactobacillus* is associated with poor performance. The members *Staphylococcus* and *Streptococcus* can be opportunistic pathogens of the intestinal tract, and these were also more abundant in IF animals; however, *Streptococcus thermophilus* has been used in probiotic supplements, and therefore these bacteria can have different effects on intestinal health depending on the species present (Mirsalami and Mirsalami, 2023) [53].

Unclassified members of the family *Lachnospiraceae* and [*Ruminococcus*] *torques group*, another member of *Lachnospiraceae*, were more abundant in the ileum (C and M) of C birds. These bacteria are known to be important producers of short-chain fatty acids like butyrate (Pryde et al., 2002) [54] that are beneficial to chickens in having anti-inflammatory properties and maintaining the integrity of the gut (Onrust et al., 2015) [55]. It is therefore not surprising that bacteria involved in short-chain fatty acid production would be more abundant in non-infected birds.

The family *Micrococcaceae* was found to be more abundant in the J of control birds. These bacteria are Gram-positive cocci that are frequently found in milk and cheeses and could be associated with fermentative processes (Basbas et al., 2022) [56] but are also associated with epithelial and mucosal tissues in humans (skin, small intestine, respiratory tract) (Igartua et al., 2017) [57].

*Candidatus Arthromitus* are spore-forming, Gram-positive bacteria of the phylum Firmicutes, also known as segmented filamentous bacteria, and they were found to be more abundant in the IL (C and M) of control healthy chickens. This taxon has shown a pattern of decreasing in microbiota of *Eimeria*-infected chickens across multiple studies, such as in the cecal mucosa, ileal lumen, and duodenal lumen of *E. acervulina*-infected chickens (Campos et al., 2023; Campos et al., 2024) [9,10], the cecal lumen of *E. tenella*-infected chickens (Campos et al., 2022) [12], and the ileal digesta of chickens infected with a mixture of *E. maxima* and *E. brunetti* (Feng et al., 2022) [58]. The presence of this genus is often associated with better performance in chickens (Markova et al., 2024) [49], although it has also been associated with chickens with “runting and stunting” syndrome (Lages de Silva at al., 2024) [59]. Its function is unknown at this point. Abundance of bacterial taxa in the intestine of poultry can be affected by other parameters besides infection, such as age, environmental conditions, feed and is therefore highly variable; however, additional studies should be able to distill the true effect of coccidia infection on the intestinal microbiota.

To connect the relative abundance of bacteria to metabolic processes they may be involved in, we carried out a predictive analysis. Although the accuracy of these analyses could be limited since the database is primarily composed of metabolic processes that occur in mammalian hosts (Sun et al., 2020) [60], the information gathered could be useful when planning future physiological and biochemical studies on the effect of microbiota on gut health. There was some but not a lot of overlap between the metabolic processes and sections of the small intestine (J or IL) and their C or M. It was previously found that metabolic pathways that could be involved in short-chain fatty acid production in the duodenum, jejunum, and ceca were reduced in birds infected with *E. acervulina* (Campos et al., 2024) [10]. This was not found to be the case in this study in which birds were infected with *E. maxima*, which are related but cause somewhat different symptomology. In the J-C of IF birds, metabolic pathways related to sugar and amino acid degradation were more abundant, and in the IL-M, the predominant pathways in IF birds were related to nucleotide biosynthesis. At this point, the significance of these findings is unknown but should be investigated in future physiological studies on the effects of *Eimeria* infection on the intestinal tract.

## 5. Conclusions

In conclusion, a symptomatic *E. maxima* infection was induced that lowered performance parameters such as BWG and FCR, as well as the morphology of the small intestine. The infection resulted in significantly decreased levels of carotenoids in the plasma, indicating loss of gut integrity. The effect of the infection was followed for 14 days PI, and its effect on the microbiota in the small intestine (J and IL) was analyzed. The infection affected alpha and beta diversity, particularly at the height of infection. The analysis of relative abundance of bacterial taxa suggested that butyrate-producing bacteria may be greater in C animals. *Lactobacillus* species were more abundant in IF animals, which is a paradoxical finding. It was predicted that pathways involved in sugar and amino acid degradation and nucleotide synthesis were more abundant in the microbiota of IF birds. It was also clear that the luminal and mucosal populations of bacteria differed, and therefore including a separate analysis of gut contents and scrapings produces a more complete picture of the ecology of the gut microbiota.

## Figures and Tables

**Table 1:** Sequencing summary of the datasets generated from microbiota samples (JC, J-M, IL-C, IL-M) processed through QIIME 2.

	J-C	J-M	IL-C	IL-M
Number of samples	60	60	60	60
Raw reads	6,321,969	1,906,686	8,520,563	3,669,527
DADA2 --p-trunc-len-f	269	235	269	271
DADA2 --p-trunc-len-r	217	205	229	208
Reads after DADA2	5,212,955	1,312,059	6,443,444	2,663,546
Reads after filtering	5,060,831	1,158,586	6,434,195	2,652,074
Reads per sample (range)	114–256,509	81–255,261	13,847–257,570	197–503,642
Mean reads per sample	84,347	19,310	107,237	44,201
Total number of ASVs	1001	1273	984	1371
ASV read length (range)	269–465	234–427	269–473	271–466
Mean ASV read length	418	358	420	385
Sequencing depth	27,462	5658	49,158	13,067

**Table 2:** Growth performance data of infected and uninfected broiler chickens ^1^.

Day Post-Infection Infection Status		BW (kg)	BWG ^2^ (kg)	FI (kg)	FCR
3 PI	C	1.12	0.23 ^f^	1.36	1.49 ^c^
	IF	1.17	0.22 ^f^	1.37	1.55 ^c^
5 PI	C	1.23	0.38 ^e^	2.40	1.58 ^c^
	IF	1.24	0.37 ^e^	2.30	1.57 ^c^
7 PI	C	1.33	0.61 ^d^	3.72	1.54 ^c^
	IF	1.50	0.45 ^e^	3.77	2.13 ^ab^
10 PI	C	1.51	0.78 ^c^	5.77	1.85 ^abc^
	IF	2.05	0.63 ^d^	5.51	2.27 ^a^
14 PI	C	1.95	1.19 ^a^	7.85	1.65 ^bc^
	IF	2.05	1.03 ^b^	7.27	1.79 ^abc^
SEM ^3^		0.12	0.03	0.29	0.11
Main effect day PI					
3 PI		1.15 ^b^	0.23 ^e^	1.36 ^e^	1.52 ^b^
5 PI		1.23 ^b^	0.37 ^d^	2.35 ^d^	1.57 ^b^
7 PI		1.41 ^b^	0.53 ^c^	3.75 ^c^	1.83 ^ab^
10 PI		1.78 ^a^	0.70 ^b^	5.64 ^b^	2.06 ^a^
14 PI		2.00 ^a^	1.11 ^a^	7.56 ^a^	1.72 ^b^
SEM ^3^		0.08	0.02	0.20	0.08
Main effect infection status					
C		1.43 ^b^	0.64 ^a^	4.22	1.62 ^b^
IF		1.60 ^a^	0.54 ^b^	4.04	1.86 ^a^
SEM ^3^		0.05	0.01	0.13	0.05
Analysis of variance		Probabilities ^4^			
Day post-infection × infection status		0.18	0.0053	0.82	*0.0487*
Day post-infection		*<0.0001*	*<0.0001*	*<0.0001*	*0.0001*
Infection status		*0.0246*	*<0.0001*	0.33	*0.0015*

^1^ Each value represents the least-square means from 6 replicate cages with 4 male chickens/cage. Chickens at 21 d of age were infected with 1000 *Eimeria maxima* oocysts (IF) or sham infected with sterile water (C) and evaluated at 14 d post-infection (PI). ^2^ Body weight gain is calculated starting at 0 PI. On 0 PI, average body weight = 0.89 kg. ^3^ SEM = pooled standard error. ^4^ Italicized *p*-values are statistically significant (*p* < 0.05). ^a–f^ Means in the same column with different superscripts are significantly different (*p* < 0.05) based on Tukey HSD mean separation.

## Data Availability

The 16S rRNA gene sequences determined in this study were deposited in the NCBI Sequence Read Archive (SRA) database (SRA accession # PRJNA1122195).
